# Human fibulin-3 protein variant expresses anti-cancer effects in the malignant glioma extracellular compartment in intracranial xenograft models

**DOI:** 10.18632/oncotarget.22344

**Published:** 2017-11-09

**Authors:** Yanyan Li, Yuan Hu, Chuanjin Liu, Qingyue Wang, Xiaoxiao Han, Yong Han, Xue-Shun Xie, Xiong-Hui Chen, Xiang Li, Eric R. Siegel, Kambiz Afrasiabi, Mark E. Linskey, You-Xin Zhou, Yi-Hong Zhou

**Affiliations:** ^1^ Neurosurgery & Brain and Nerve Research Laboratory; ^2^ Department of Emergency Surgery, The First Affiliated Hospital of Soochow University, Suzhou, Jiangsu, China; ^3^ Neuroepigenetic Reseach Lab, Queensland Brain Institute, The University of Queensland, St Lucia, QLD, Australia; ^4^ Department of Biostatistics, University of Arkansas for Medical Sciences, Little Rock, AR, USA; ^5^ Brain Tumor Research Laboratory, Department of Surgery, University of California Irvine, Irvine, CA, USA; ^6^ Ziren Research, Irvine, CA, USA

**Keywords:** protein therapeutics, GBM, tumor heterogeneity in vivo model, EGFR/NOTHC signaling, MMP2

## Abstract

**Background:**

Decades of cytotoxic and more recently immunotherapy treatments for malignant glioma have had limited success due to dynamic intra-tumoral heterogeneity. The dynamic interplay of cancer cell subpopulations has been found to be under the control of proteins in the cancer microenvironment. EGF-containing fibulin-like extracellular matrix protein (EFEMP1) (also fibulin-3) has the multiple functions of suppressing cancer growth and angiogenesis, while promoting cancer cell invasion. EFEMP1-derived tumor suppressor protein (ETSP) retains EFEMP1’s anti-growth and anti-angiogenic functions while actually inhibiting cancer cell invasion.

**Methods:**

In this study, we examined the therapeutic effect on glioblastoma multiforme (GBM) of an *in vitro* synthesized protein, ZR30, which is based on the sequence of ETSP, excluding the signaling peptide.

**Results:**

ZR30 showed the same effects as ETSP in blocking EGFR/NOTCH/AKT signaling pathways, when applied to cultures of multiple GBM cell lines and primary cultures. ZR30’s inhibition of MMP2 activation was shown not only for GBM cells, but also for other types of cancer cells having overexpression of MMP2. A significant improvement in survival of mice with orthotopic human GBM xenografts was observed after a single, intra-tumoral injection of ZR30. Using a model mimicking the intra-tumoral heterogeneity of GBM with cell subpopulations carrying different invasive and proliferative phenotypes, we demonstrated an equal and simultaneous tumor suppressive effect of ZR30 on both tumor cell subpopulations, with suppression of *FOXM1* and activation of *SEMA3B* expressions in the xenografts.

**Conclusion:**

Overall, the data support a complementary pleiotrophic therapeutic effect of ZR30 acting in the extracellular compartment of GBM.

## INTRODUCTION

GBM is a deadly form of brain cancer, for which there has been marginal improvement on survival despite 40 years of research/clinical trials. It is coming to be recognized that the challenge in treating a solid tumor, such as GBM, is the inability to eliminate invading cancer cells by surgical resection and chemo/radiation therapy. Surviving invasive stem-like tumor-initiating cells (STIC) form recurrent tumor at the original tumor site or move into new sites, where they establish their tumor microenvironment and re-form a heterogeneous tumor population, including STIC and fast-growing tumor-mass forming cells (TMC). Although most GBMs are found to have over-expression of cell membrane receptor EGFR, pro-angiogenic protein VEGFA, and pro-invasive protein MMP2, there has been no success in improving survival with drugs targeting MMP2, EGFR and VEGFA alone or in combination [1, 2]. Invasive STIC in GBM are commonly found to lack the high expression of EGFR shown in TMC subpopulations, but to have activation of NOTCH signaling, which has been demonstrated to maintain “stemness” [3] and other features associated with cancer stem cells and their resistance to radiation therapy [4]. However, currently there is no report of success in treating GBM by targeting NOTCH signaling alone.

The poor success in treating GBM is due to failure to effectively disrupt the powerful and puzzling interplay among the highly proliferative and highly invasive tumor cell subpopulations and the tumor microenvironment. In an effort to develop a therapeutic agent to meet this unmet need, Zhou et al. dissected and engineered EGF-containing fibulin-like extracellular matrix protein (EFEMP1)’s functional modules/domains and created the EFEMP1-derived tumor suppressive protein (ETSP) that inhibits key oncogenic signaling pathways (e.g. EGFR, NOTCH signaling pathways) differentially activated by GBM functional cell subpopulations and their interaction with the tumor-promoting microenvironment (e.g. MMP2) [5].

EFEMP1, also known as fibulin-3, was originally known as a protein in senescence with angiostatic function. It was later reported widely in solid tumors, mostly with hyper-methylation, repressed expression, and tumor suppression functions, but sometimes with a tumor-promoting role (see references in review [6]). Dual functions of EFEMP1 was reported in two key tumor cell subpopulations carrying features of STIC and TMC [7]. ETSP was created by removing the pro-invasive C-terminal fibulin domain from EFEMP1 and introducing a point mutation that turned a weak integrin-binding site in one of the five EGF-like modules into a strong one. Compared to EFEMP1’s role in GBM, ETSP has enhanced effect on antagonizing EGFR signaling and gained novel functions of anti-NOTCH1, anti-MMP2, and anti-cell invasiveness.

Like EFEMP1 [8], the complementary pleiotrophic tumor suppressive effect of ETSP should be exerted in the tumor’s extracellular compartment. It could become a novel and effective cytostatic cancer treatment through direct access to the extracellular matrix compartment to suppress cancer growth. To prove this hypothesis, an *in vitro* synthesized protein, named ZR30, which is based on ETSP’s sequence but lacks the *N*-terminal signal peptide, which directs the protein’s extracellular exportation, was applied directly to cell cultures and GBM xenografts via intratumoral (i.t.) injection into xenografts formed in nude mice brains. The effects on known targets of ETSP on GBM cells and their growth *in vivo* were studied using multiple GBM-derived cell lines and primary cultures, as well as high MMP2-expressing cell lines of cervical cancer, stroma of prostate cancer and metastatic prostate cancer, and three orthotopic GBM xenograft models. New targets for anti-cancer effects of ZR30 on GBM cells were further explored by this study.

## RESULTS

### ZR30 acts on the same targets of ETSP

**Figure 1:**
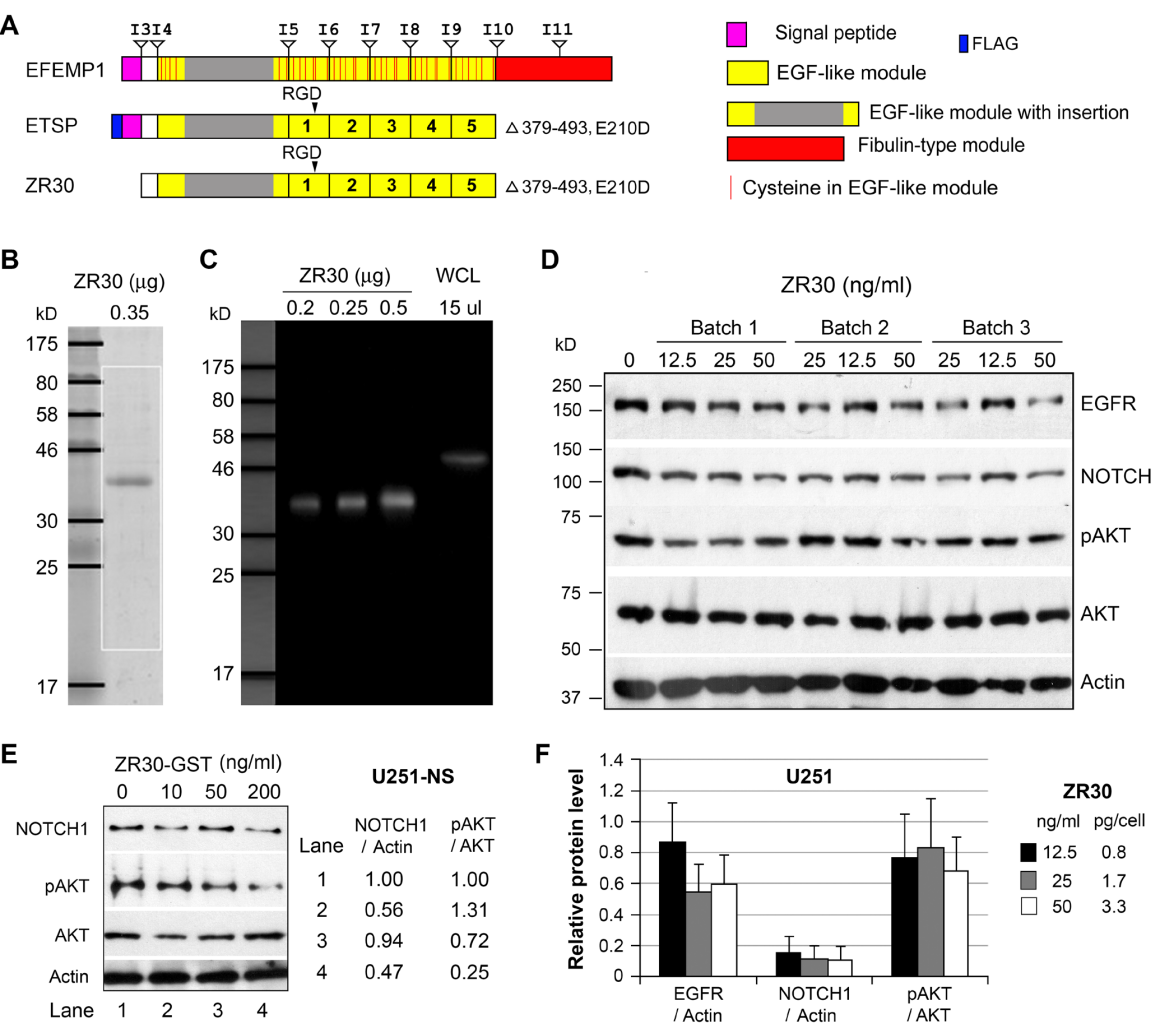
The bioactivity and reproducibility of ZR30 **A**. Alignments of protein functional domains of EFEMP1, ETSP, and ZR30, with boundaries of exons and introns shown above EFEMP1. **B**. Coomassie Blue-staining of ZR30. **C**. Immunoblotting of ZR30 with EFEMP1 antibody. Positive control was a whole cell lysate (WCL) of 293 cells transfected with an EFEMP1 expression vector. **D**. Immunoblot of U251-NS treated with GST-tagged ZR30 for 2 days in culture. **E**. Immunoblot of U251 cells treated with three different batches of ZR30 at various concentrations (70, 140, 180 ng/ml) for 4 days, followed by a 2-day serum starvation. **F**. Densitometry of immunoblot shown in panel D, with Blank treatment (0 ng ZR30) set at unity. Bar heights and error bars are averages and standard errors, respectively, of cells treated with three different batches of ZR30, in comparison to untreated cells.

ZR30 used in this study was provided by Ziren Research, LLC, produced by an *in vitro* cell-free system based on ETSP but excluding the signal peptide (Figure 1A), fused to GST tag for purification, has a size of 38.61 kDa after GST removal in SDS-PAGE gel (Figure 1B) and detectable in immunoblotting by an antibody for human EFEMP1 (Figure 1C).

GBM cell line U251-NS, which was enriched with STIC with high expression of NOTCH1 and barely detectable EGFR [7], was examined using ZR30 prior to removal of GST. As shown in Figure 1D, NOTCH1 (normalized to Actin) in U251-NS was reduced by about half following a 2-day treatment with GST-ZR30 (ZR30 without removal of GST tag) at high dosage (200 ng/ml), and pAKT (normalized to AKT) level was further reduced (a decrease of 75%).

With the biological activity of GST-ZR30 thus proven, three batches of ZR30 (with removal of GST) were examined for production reproducibility, which is critical for future clinical application. A 2-day treatment with low dosages of ZR30 from three batches was carried out in U251 cells expressing both NOTCH and EGFR. As shown in Figure 1E and 1F, ZR30 from different batches showed a strong suppressive effect on NOTCH1 (a decrease of 84-89%) at all three low dosages (similar to that in experiments shown later). It showed no effect on EGFR and AKT phosphorylation except for minor decreases (40%+/-20% and 30%+/-25%), respectively, at dose of 50 ng /ml, compared to an un-treated control. ZR30 was expected to have little effect on EGFR and AKT phosphorylation without stimulation by EGF.

**Figure 2:**
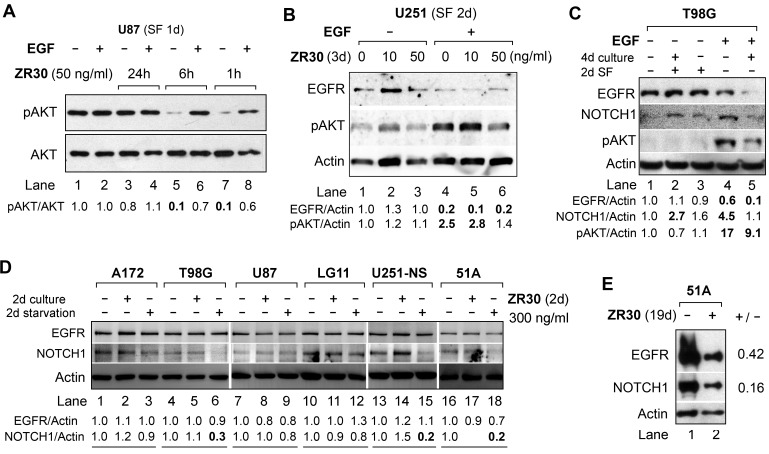
Effect of ZR30 on EGF-mediated activation of EGFR signaling and NOTCH1 expression by immunobloting **A**. U87 cell cultures with or without ZR30 for 1h – 24 hours, followed by a 1-day serum free (SF) culture. **B**. U251 cell cultures with or without ZR30 for 3 days, followed by a 2-day SF culture. **C**. T89G cell culture with or without ZR30 (100 ng/ml) for 4 days, followed by a 2-day SF culture. EGF was added for 30 min at a concentration of 50 ng/ml prior to harvesting cells for studies shown in panels A-C. **D**. Six GBM cell lines cultured in medium supplemented with serum (A172, T98G, U87, LG11) or with EGF, bFGF, and B27 (U251-NS, 51A) for two days, followed by a 2-day culture in basal medium (starvation of serum or growth factors). **E**. 51A culture with or without ZR30 (100 ng/ml) for a total of 19 days, with the medium changed at 5-day intervals. Densitometry of untreated cells was set in unity to compare with ZR30-treated cells.

The effects of ZR30 in targeting the EGFR/NOTCH/AKT signaling pathways were further examined in multiple high-grade GBM cell lines and a GBM-derived primary culture 51A. ZR30 contains all five EGF modules of EFEMP1, which may have a ligand-like effect in targeting AKT signaling through EGFR. Hence an initial test of the protein’s ligand function was carried out over a few hours of treatment. As shown in Figure 2A (lanes 5, 7), a short-term (1-6 h) effect of ZR30 in reducing the pAKT level was observed in U87 cells, consistent with ZR30’s ligand-like effect on EGFR. The short-term effect of ZR30 on reducing AKT phosphorylation was re-activated by EGF (Figure 2A, lanes 6, 8). Further experiments were carried out to examine ZR30’s long-term effect on disabling EGFR-activation in response to EGF.

The canonical process of EGF-mediated activation of EGFR includes EGFR internalization, hence reduction of membrane level of EGFR, and increase of AKT phosphorylation (pAKT), which normally occur in synchronized cells by serum starvation for 1-2 days. In U251 cells after serum starvation for 2 days, exposure of cells to EGF (50 ng/ml) for 30 min showed EGFR/AKT activation, whereas adding human recombinant EFEMP1 or induction of ectopic EFEMP1 or ETSP over-expression blocked EGFR activation [5, 8, 9]. Using the same conditions we analyzed ZR30-treated U251. The result showed EGF-mediated activation of EGFR signaling in un-treated cells (Figure 2B lanes 1 and 4). In ZR30-treated cells at higher dose (50 ng/ml), not at lower dose (10 ng/ml), EGF-activation of pAKT was inhibited by 44% (Figure 2B lanes 4 and 6).

The same effect of ZR30 on blocking EGFR signaling was also seen in malignant glioma cell line T98G. In contrast to U251, an increase of NOTCH1 was observed in T98G in response to EGF (50 ng/ml for 30 min), while ZR30 countered this effect (Figure 2C, lanes 1, 4, and 5). In addition, in T98G, ZR30 sensitized EGF-mediated EGFR degradation (Figure 2C, lanes 4 and 5), which was not observed in U251 (Figure 2B, lanes 4 and 6). The result from ZR30 on increasing NOTCH1 in T98G shown in Figure 2C (Lanes 1-3) was not shown in a repeated experiment and assay (Figure 2D, lanes 4 and 5), rendering experimental error to be verified.

We then further studied ZR30’s effect on NOTCH1 expression in six cell lines of GBM, under active (in the first two days) or slow (the second two days) growth stages, controlled by their growth medium with, or without, serum supplementation (A172, T98G, U87, LG11) or growth factors (U251-NS, 51A), respectively. As shown in Figure 2D, when ZR30 was added to cells in the first 2-day culture in their growth-supporting culture conditions followed by 2-day culture in basal medium, NOTCH1 levels were unchanged in all lines of cells (lanes 2, 5, 8, 11, 17), except U251-NS (lane 14), where NOTCH1 level was slightly increased, compared to untreated control (lanes 1, 4, 7, 10, 13, 16). However, when given in the second 2-day culture under slow growth conditions, NOTCH1 levels were greatly reduced (a decrease of 70-80%) in T98G, U251-NS, and 51A, and a minor reduction (by 20-30%) in A172, U87, and LG11 happened, compared to untreated cells. In contrast, EGFR levels were unchanged by ZR30 given in both time periods, except for a minor reduction (a decrease of 20-30%) shown in U87 and 51A.

In GBM-derived stem-like cell-enriched 51A, established and maintained in DMEM/F12 containing growth factors including EGF, the anti-EGFR and anti-NOTCH effects were shown after a long-term exposure to ZR30, with reductions of both EGFR and NOTCH1 (a decrease of 58% and 82%, respectively) levels, compared to the untreated cells (Figure 2E).

### ZR30 inhibits the activation of MMP2 over-expressed by cancer cells

**Figure 3:**
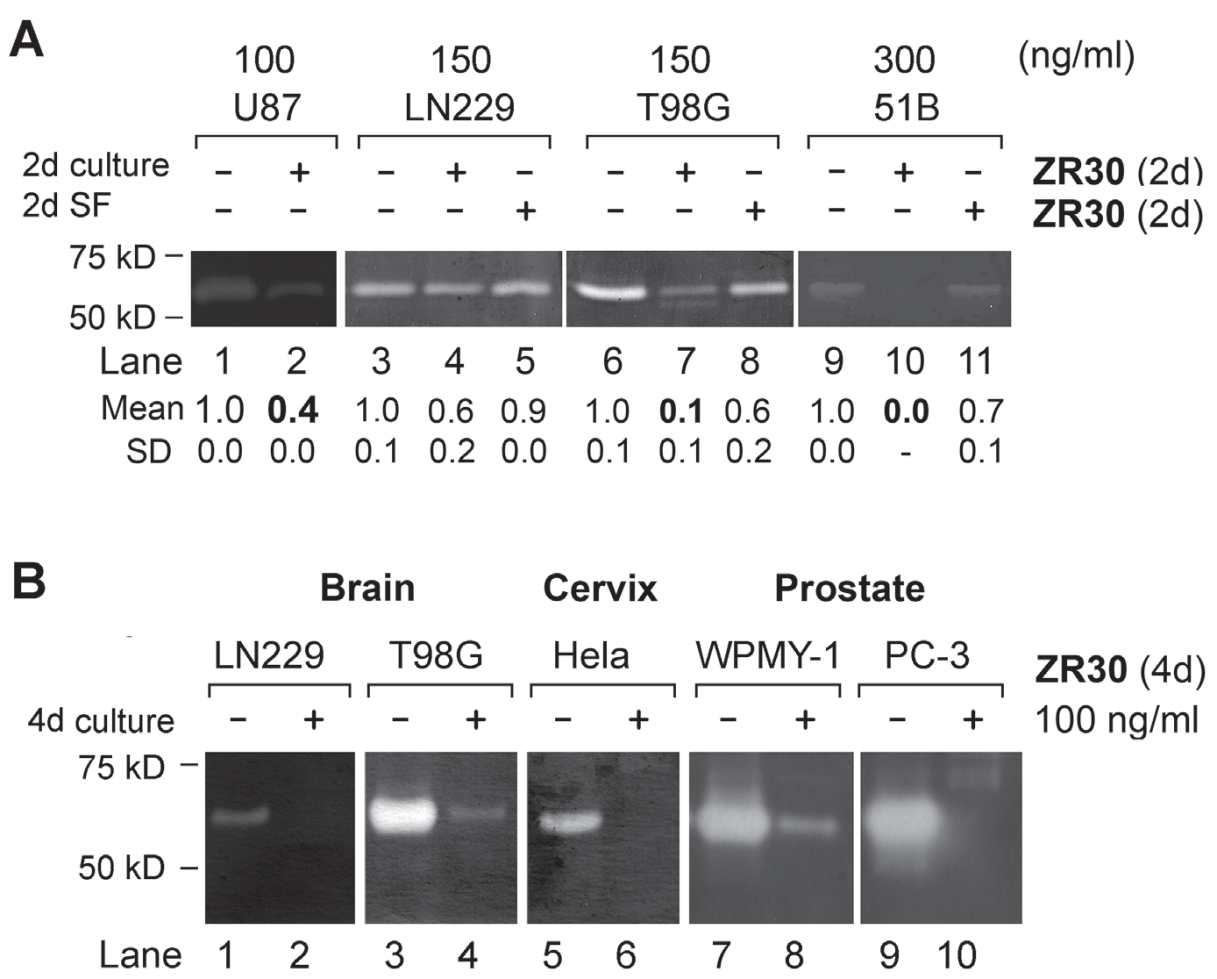
Effect of ZR30 on MMP2 activation by Gelatin zymography **A**. Equal amount of protein precipitated from 2-day conditioned medium of various cell lines of GBM treated by ZR30, as described in Figure 2D. Mean and SD of densitometry of MMP2 from repeated assays are shown, with untreated cells set in unity. **B**. Gelatin zymography assay of ZR30-treated cancer cells originating from brain, cervix and prostate.

Over-expression of matrix metallopeptidase 2 (MMP2) and its extracellular activation and pro-invasion function have been widely reported in cancer, including malignant glioma [10, 11]. Using the gelatin zymography assay for quantification of the level of active gelatinase (here MMP2 based on the size of 67 kD) in conditioned medium of cell culture (see supplementary text for the methods details), we analyzed the effect of ZR30 on the regulation of MMP2 activation in three high-MMP2-expressing GBM cell lines (LN229, U87, and T98G) and a GBM primary culture (51B). As shown in Figure 3A, following a 2-day culture with, and without, ZR30, active MMP2 levels were reduced in medium of cells that had continued their 2-day culture in basal medium. However, no effect of ZR30 during culture in basal medium on MMP2 was observed.

GBM cells, and cancer cells originating from other organs (e.g. prostate, cervix), having high expression of MMP2, were subjected to a 4-day culture with and without ZR30, and the proteins precipitated from 2-day conditioned medium were examined by gelatin zymography. Compared to 2-day treated cells (Figure 3A, lanes 4 and 7), cells subjected to a 4-day treatment by ZR30 showed a greater reduction in the level of extracellular activated MMP2 (Figure 3B, lanes 2 and 4). The ZR30-mediated inhibition of MMP2 activation in GBM cells was similarly observed in other human cell lines of cervical cancer, prostate cancer stroma and metastatic prostate cancer (Figure 3B, lanes 5-10). Only MMP2 was seen in the analyzed cells by gelatin zymography.

### Therapeutic efficacy of ZR30 by intra-tumoral injection with survival end-point

To prove ZR30’s therapeutic potential for GBM treatment, an animal experiment was designed with a single, 5-10 µl i.t. injection of ZR30 to avoid direct physical damage to the mouse brains from injection. We first used an orthotopic GBM xenograft model of U251, which has the 9-STR (Short Tandem Repeat) profile of U-251 MG and relatively low i.c. tumorigenicity compared to U251HF [3], to perform intra-tumoral (i.t.) injection of ZR30 10 or 21 days post cell implantation, which is estimated to be at early tumor onset or early tumor development, because median survival of mice is known to be 6-7 weeks.

**Figure 4:**
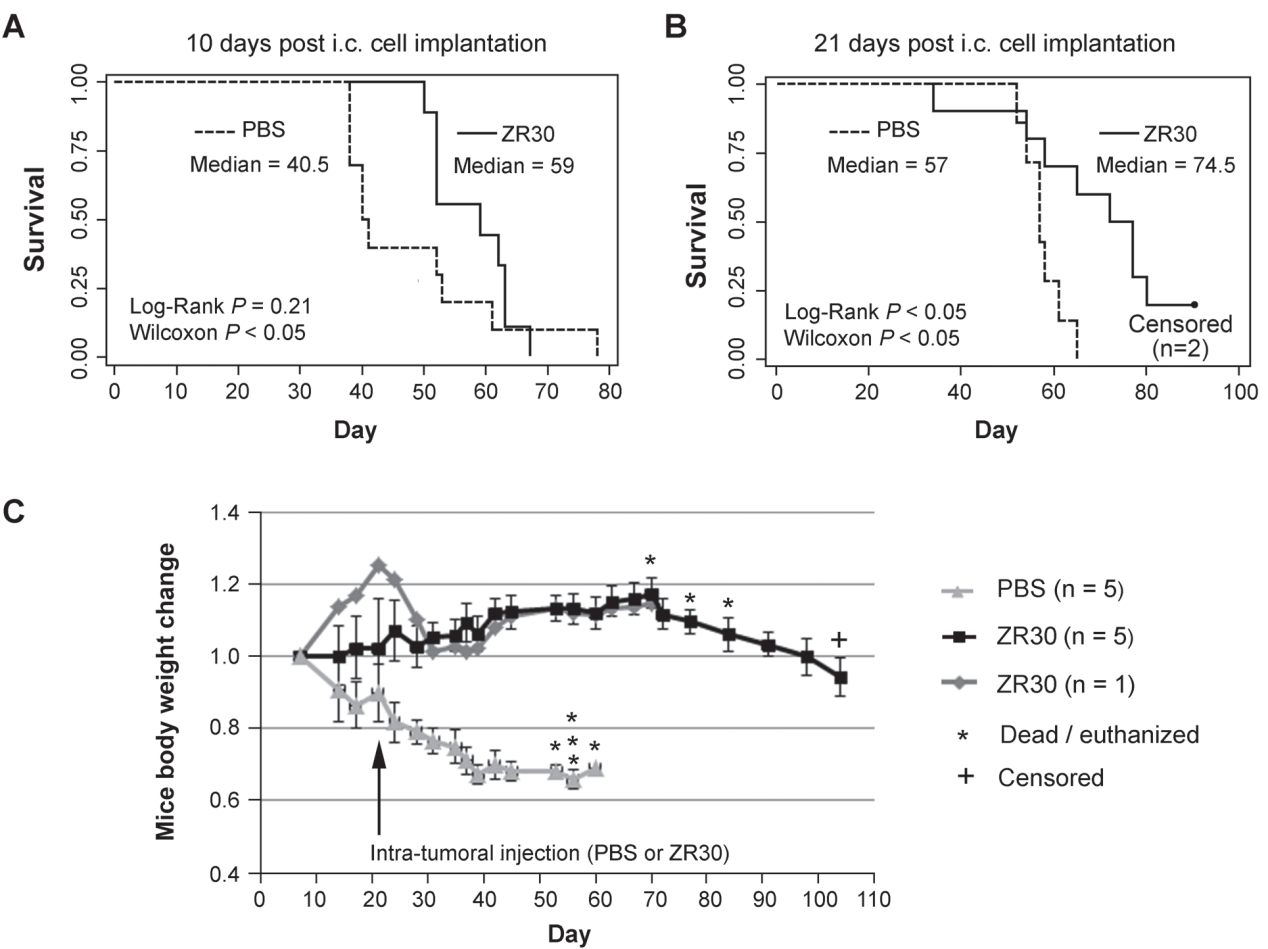
Therapeutic effect of ZR30 in the U251 xenograft model after i.t. drug administration **A** and **B**, Kaplan–Meier curves of 36 mice of similar weight and physical condition 1 week after intracranial (i.c.) tumor cell implantation (2X10^5^ cells), randomly divided into four groups (8-9 mice / group), subjected to one-time intra-tumoral (i.t.) injection of 5 µl or 10 µl PBS or ZR30 (70 ng/µl) at the time 10 or 21 days post cell implantation, respectively. Two mice in the ZR30-treatment group were euthanized 104 days post i.c. implantation, when they showed 23% and 21% weight loss, comparing to their weight 70 days post i.c. implantation. **C**. Mouse weight changes compared to that 1 week post cell implantation. Lines and bars are mean and SEM for 5 mice.

Figure 4A and 4B show the survival data of mice subjected to PBS and ZR30 treatments. Statistical analysis using Wilcoxon pair-wise comparisons showed significantly longer survival for ZR30-treated mice compared to PBS-treated mice, for both early and late-treatment time points, with median survival prolonged by 19 or 18 days (46% or 31% increase in median survival, respectively). A more stringent Cox Regression analysis confirmed the effect of ZR30 on improving survival given the 21-day post cell implantation.

Figure 4C showed changes of body weights that reflected the time course of gain and loss of therapeutic effects. Mice in the PBS control group lost 30% of their weight over a 3-week period post-injection of PBS. In contrast, mice in the ZR30 treatment group increased in weight by 1.2 fold over a 7-week period post injection of ZR30, followed by a rapid loss of weight over 1-2 weeks, which is a sign of tumor progression. One mouse in treatment group showed an increase in its body weight prior to treatment, which caused a slightly higher, but not significant, average weight of the experimental group over that of the control group prior to treatment. One mouse in the treatment group lost 20% of its weight over a 2-week period post ZR30 injection, stopped losing weight for one week, and then gained weight and maintained it over the following four weeks. It may be a case where ZR30 was injected a small distance away from the tumor on-set site, but then carried out its tumor suppression effect when tumor cells reached the area where the drug had altered the tumor-supporting microenvironment.

### ZR30 targets both TMC and STIC subpopulations of U251 in an orthotopic tumor model

The *in vitro* studies described above demonstrated multiple targets of ZR30 in the suppression of both tumor-mass-forming cells (TMC) involving activation of EGFR/AKT signaling, and stem-like tumor-initiating cells (STIC) involving activation of NOTCH signaling. TMC and STIC subpopulations were enriched in U251 and U251-NS, respectively, and the two tumor cell subpopulations differentially contributed to i.c. xenograft formation, as demonstrated in Hu et al. 2013 [12]. We then performed an animal experiment by i.c. co-implantation of U251-GFP and U251NS-RFP cell mixtures (1:1 ratio, total 1X10^5^ cells). A week later, the same volume (10 µl) of ZR30 at three concentrations (18, 70, and 180 ng/µl) or PBS were injected through the hole and at the same depth as for cell implantation. According to information on animal weight loss, the time of treatment appeared to be at the stage of aggressive tumor development. All animals in the control and treatment groups (13-16 mice/group) were euthanized 8-9 days following i.t. injection. The entire brains were removed, and the right hemispheres where tumor cells were implanted were cut out to extract DNA.

**Figure 5:**
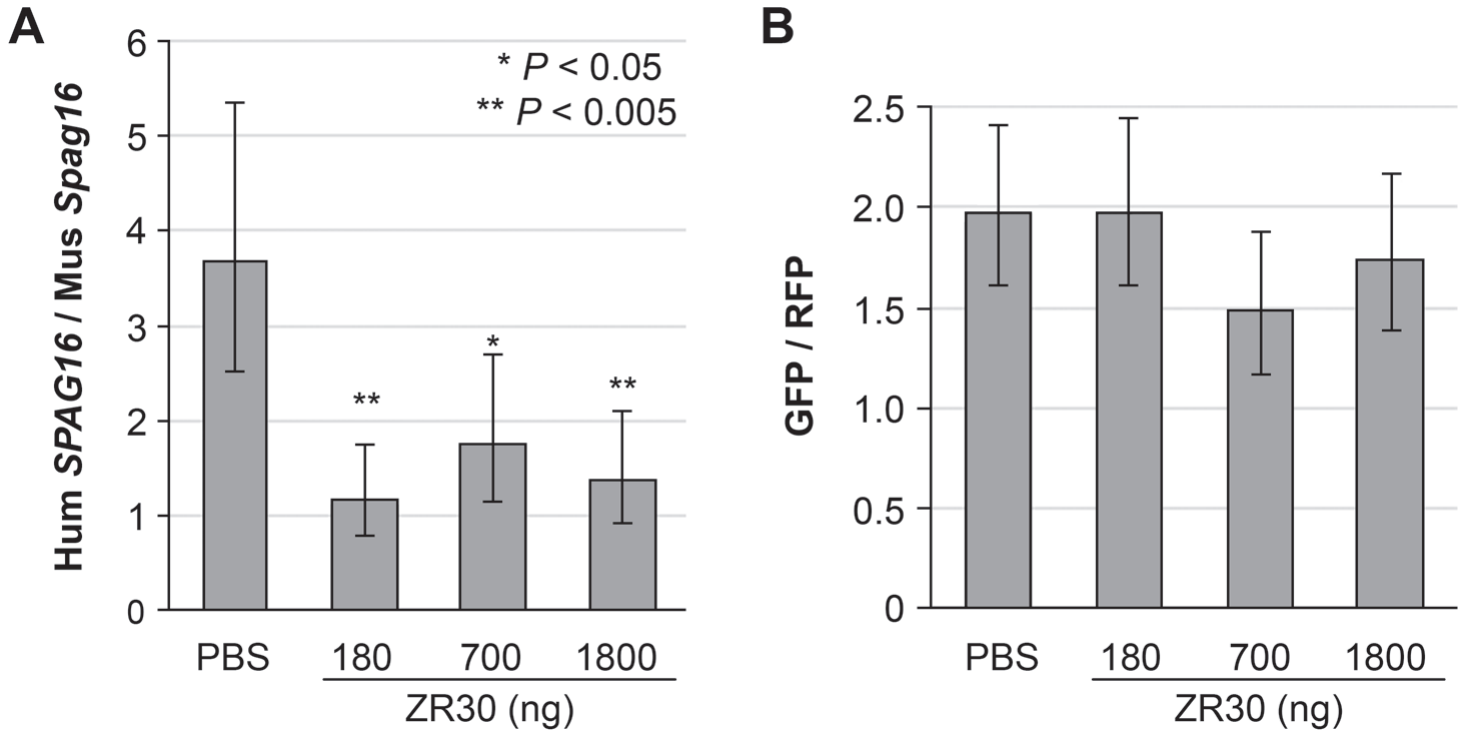
Effect of ZR30 on *in vivo* growth of both STIC and TMC subpopulations of U251 **A**. comparative quantitative (CQ)-PCR assay quantified the effect of ZR30 on overall tumor growth in an orthotopic model using a U251-GFP and U251NS-RFP mixture (1:1 ratio, total 1X10^5^ cells), with DNA samples extracted from the entire right hemisphere of mice euthanized 15-16 days post i.c. cell implantation, and 8-9 days post intra-tumor (i.t.) injection of 10 µl PBS or ZR30 at various levels, by comparison of the human *SPAG16* and mouse *Spag16* gene copy number ratios. **B**. comparison of GFP to RFP gene copy number ratios in DNA samples described above. Bar heights and error bars are averages and 95% confidence intervals. Significant Bonferroni-adjusted *P* values from comparisons to the PBS-control are shown.

We used comparative quantification (CQ) of human and mouse genes by real-time PCR on DNA with human- or mouse-gene-specific primers on the single-copy gene *SPAG16* of human or *Spag16* of mouse. The accuracy and high efficiency of CQ-PCR was shown in detection of 1p19q loss in gliomas [13]. To avoid any bias in sampling, we extracted DNA from the entire right hemisphere of the brain where tumor cells were implanted. As shown in Figure 5A, an average 3.7 ratio of Human (Hum) *SPAG16* / mouse (Mus) *Spag16* DNA copies in PBS-treated mice indicated that large volumes of human GBM xenografts had grown in the mouse right hemispheres. The ratios of Hum *SPAG16* / Mus *Spag16* DNA copy were significantly reduced to average levels of 1.2, 1.7, and 1.4 in ZR30-treated mice at 180, 700, and 1800 ng dosages, respectively. The absence of a dose-dependent suppression effect on the overall tumor growth could be due to the lowest dose having achieved as great an effect as the highest dose in terms of altering the local environment to suppress the growth of the nearby tumor cells.

CQ-PCR was also applied to determine *in vivo* growth of two tumor cell subpopulations, here the ratio of U251 and U251-NS, which were differentially transduced with lentiviral vectors carrying GFP and RFP-coding DNA, respectively. As shown in Figure 5B, the ratio of GFP to RFP copy numbers in xenografts lacked a significant difference between control and ZR30-treated mice. The result showed that the tumor suppression effect from ZR30 applied equally to TMC and STIC subpopulations of U251.

### ZR30 targets *in vivo* expression of *SEMA3B, FOXM1*, and *IGFBP3*

**Figure 6:**
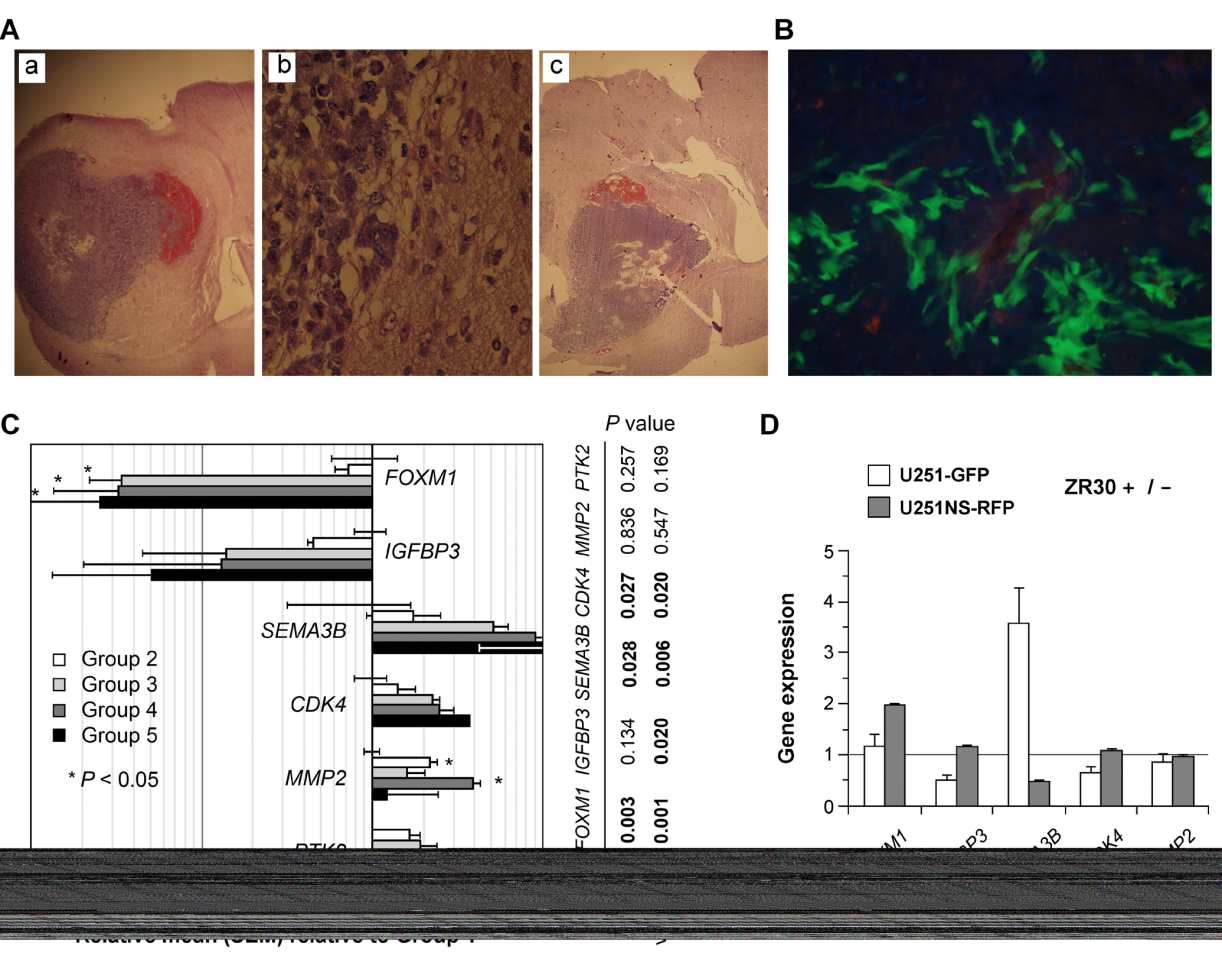
Effect of ZR30 in Cannula-guided i.c. U251 xenograft with i.t. drug delivery with *in vivo* regulation of *FOXM1, IGFBP3* and *SEMA3B* expressions **A**. Representative pictures of H&E staining of mouse brains showing tumor burdens following intracranial implantation via guide cannula, of cell mixtures of U251-GFP and U251NS-RFP (1:9 ratio, total 1X10^5^ cells), 10 days after intra-tumor (i.t.) injection of 5 µl PBS (panels a-b) or ZR30 (180 ng/µl) (panel c). **B**. Representative fluorescence picture of OTC-embedded tissue of the PBS-treated mouse described above. **C**. Comparison of gene expressions quantified by real-time qRT-PCR with human-gene-specific primers in RNA samples of i.c. U251 xenografts derived from cell mixtures of U251-GFP and U251NS-RFP (1:9 ratio), normalized to *ACTB*. The mean of gene expressions in two PBS-control mice for single dose treatment, which was set at unity (Group 1), was compared to that of two single dose ZR30-treated mice having no improvement in survival (Group 2), three single dose ZR30-treated mice with 1-wk improvement of survival (Group 3), two control mice for second treatment (Group 4) and two double dose ZR30-treated mice (Group 5). **D**, quantification of gene expressions by both cell lines *in vitro* after 3 days of culturing in medium containing ZR30 (150 ng/ml). Error bars show SD in repeated quantification.

We performed a third animal experiment with the orthotopic model of U251 showing tumor heterogeneity (mixture of U251-GFP and U251NS-RFP, 1:9 ratio, total 1X10^5^ cells) using cannula-guided injection of cells and treatments (5 µl PBS or ZR30, 180 ng/ µl) 10 days post cell implantation. Forty-seven mice with cannula implants were used to inject cells. Only one mouse died, 7 days post cell implantation, which was excluded in the survival analysis. Median survival of mice in the control group (PBS treatment, n = 14) was 30.5 days, with small variation (SD = 5 days, SEM = 0.16 day). Twenty-three (72%) mice in the ZR30 treatment group (n = 32) survived beyond the median survival time of mice in the PBS-control group, and fifteen (47%) survived beyond the longest surviving mouse in the PBS-control group. The result of this animal experiment independently validated the therapeutic effect of ZR30 in treating U251 xenografts with low i.c. tumorigenicity via one dose i.t. injection, as shown above in Figure 4. Figure 6A shows the histology of the xenograft from PBS (panels a and b) and ZR30 (panel c) treatments. The track of the cannula needle and necrotic tumor area at the end of the needle were shown in panel c. In the formed xenografts, as shown in Figure 6B, the majority tumor cells were U251 expressing GFP, while RFP-expressing U251-NS cells were in the minority and in areas of blood vessels, as previously reported for this GBM xenograft model [12].

The thirteen mice that lived for 42 days after i.c. implantation of tumor cells with i.t. injection of ZR30 10 days post cell implantation were divided into two groups to receive a 2^nd^ treatment with 5ul PBS or ZR30 (180 ng/ µl). However, all mice in both groups showed severe weight loss 1 week after the 2^nd^ treatment, including four that died 1-2 days post the second i.t. treatment. All remaining mice were euthanized 10 days after the 2^nd^ treatment, and their brains were removed for histology or RNA analysis. One mouse with two doses of ZR30 treatment showed no sign of weight loss and CQ-PCR of the entire right hemisphere showed human SPAG16 / mouse Spag16 gene copy ratio = 0.03, suggesting successful cell implantation and ZR30-mediated suppression of tumor formation from both i.t. treatments.

With RNA samples extracted from the entire right hemispheres of mice from control and ZR30-treament groups, we examined a set of genes with effects on proliferation, invasion and angiogenesis with human-specific PCR primers and standards established and provided by Ziren Research (Irvine CA, USA). Gene expression data, after normalization to *ACTB*, were analyzed by Welch’s *t*-test, to compare PBS- and ZR30-treated tumors (Group 1, n=2) for an *in vivo* effect of ZR30. Single ZR30-treated tumors were grouped into two, for mice without (Group 2, n=2) or with (Group 3, n=3) improvement in survival. Tumors from second treatments of PBS and ZR30-treated tumors were in Groups 4 (n=2) and 5 (n=2), respectively, from mice with about 3 weeks of survival improvement over the median survival of PBS-treated mice (30.5 days, SD=5 days, n=14). As shown in Figure 6C, based on significance from the t-test, there was a decrease of *FOXM1* (a decrease of 97% for all Groups 3-5) in all ZR30-treated tumors from mice with improved survival, and an increase of *MMP2* in ZR30-treated tumors from mice with or without survival improvement. High levels of decrease and increase of *IGFBP3* and ***SEMA3B***, respectively, were observed in ZR30-treated tumors from mice with improved survival, but lacked significance in the t-test due to the small sample size. T-tests were then performed between combined tumors from Groups 1 and 2 and tumors from Group 3 and combined tumors from Groups 4 and 5. Significant *P*-values (Figure 6C, right to the panel) showed a decrease of *FOXM1* and increases of *SEMA3B* and *CDK4* in mice with survival improvement (1 and 3 weeks). There were no significant changes in *PTK2* expression for any of the comparisons.

We then further examined the *in vitro* effect of ZR30 on genes which showed changes in ZR30-treated tumors, in the same cell lines (U251-GFP and U251NS-RFP) used for the animal experiment by mixing them together prior to i.c. implantation. As shown in Figure 6D, after treating the cells in culture with or without ZR30 (150 ng /ml) for three days, and normalizing to *ACTB*, the greatly downregulated *FOXM1* in tumors by i.t. ZR30-treatment was not observed in either of the *in vitro* cell cultures, with a 2-fold increase in U251NS-RFP. The downregulation of *IGFBP3* in tumors from mice with a 3-week prolonged survival (comparison of tumors from G1 and G2 with tumors from G4 and G5) was observed in U251-GPF but not in U251NS-RFP. The upregulated *SEMA3B* in tumors by i.t. ZR30-treatment was observed by a 4-fold increase in U251-GFP. In contrast, ZR30 caused a 50% reduction of *SEMA3B* expression in U251NS-RFP. Expressions of *CDK4* and *MMP2* were not affected by ZR30 in either of the cell lines *in vitro*. Since the xenografts were composed mainly of U251-GFP (see Figure 6B), the effect of ZR30 on expressions of *IGFBP3* and *SEMA3B* by U251-GFP cells in culture is consistent with that shown in xenografts.

## DISCUSSION

Data presented above showed reproducible production of an *in vitro* synthesized protein ZR30, based on the sequence of a human fibulin-3 protein variant, ETSP, omitting the *N*-terminal signal peptide for extracellular export, and its tumor suppressive effects by targeting the same oncogenic proteins (MMP2, EGFR, NOTCH1) and AKT-mediated signaling pathway in GBM cell lines, as shown by overexpression of ETSP. ZR30’s effect in reducing the level of extracellularly activated MMP2 appears to be a general effect in cancer cells, likely achieved by reducing MMP2-activating factor(s). For validating the therapeutic effect of ZR30, we used the intra-tumoral delivery route most closely resembling clinical reality and a robust GBM-representing *in vivo* model, which is the only model so far with the power to differentially show the two tumor cell subpopulations having behaviors relevant to the fast growing and high invasive natures of GBM. Overall, the data showed a therapeutic effect of ZR30 delivered by the intra-tumoral route in three different U251 orthotopic models, with a demonstration of survival improvement and also suppression of the growth of both TMC and STIC *in vivo*.

This study identified two potential new tumor suppression mechanisms of ZR30 not studied yet in ETSP-expressing GBM cells and their xenografts: 1) up-regulation of *SEMA3B*, which is a tumor suppressor gene for lung cancer, breast cancer, renal cancers, ovarian tumors, esophageal squamous cell carcinoma, and liver cancer [14-16], and 2) down-regulation of *FOXM1*, which is an oncogene with major roles in tumor growth, angiogenesis, invasion and metastasis [17]. The effects of having these two genes up- and down-regulated by ZR30 require further study, especially on the cell-context-dependent differential regulation of *SEMA3B* expression in U251 and U251-NS lines, given the suggested contradictory role of SEMA3B in gliomas [18]. The *in vivo*-specific down-regulation of *FOXM1* suggests that ZR30 has the ability to alter the tumor micro-environmental cue to which the transcription of *FOXM1* indirectly responds, which may result in a lower expression of *IGFBP3* comparing to control, due to a less hypoxic environment [19].

Overall, the current data show multiple targets of ZR30 for its tumor suppressive effect, including membrane receptors (EGFR, NOTCH1) and their downstream AKT-signaling, pro-invasive and pro-angiogenic extracellular protease (MMP2), and oncogenic transcription factor (FOXM1). ZR30-mediated reactivation of tumor suppressor gene *SEMA3B* expression may provide a microenvironment which further suppresses tumor growth through its angiostatic function. It is noted that these simultaneously regulated genes/proteins/signaling pathways may work together to achieve the observed therapeutic effect in the human orthotopic glioma model of nude mice. The fact that ZR30 is derived from a normal human extracellular matrix protein has the added potential advantage of lowering the risk of toxicity or immuno-sensitization, while its site of action in the extracellular compartment greatly simplifies treatment delivery issues, as intracellular incorporation is not required. Patients are expected to benefit from local delivery of ETSP to suppress the regrowth of GBM by tumor cells infiltrated into surrounding tissues. With design of proper drug delivery mechanisms, and perfection of an optimal dosing level and schedule, an effective therapy for GBM could emerge after many years of stagnation in this field.

## MATERIALS AND METHODS

### Human cell lines

Human cell lines derived from GBM (two variants of U251, U87, A172, LG11, LN229, T98G, and U251-NS, which is STIC of U251), syngeneic primary cultures of GBM (51A with EGFR double minutes and 51B without EGFR double minutes [20]), and human cancer cell lines of cervix (Hela) and prostate (PC-3 and WPMY-1) were used in this study. Information about cell line origin, authentication, mycoplasma testing, and information about cell lines and their culture conditions are described below.

The human glioblastoma cell line U251 and U87 were purchased from the Cell Bank Type Culture Collection of the Chinese Academy of Sciences (CBTC-CCAS, Shanghai, China) in November 2014, which were imported by Institute of Cell Biology, CAS from RIKEN Cell Bank of Japan in 2005 and 2009, and passed STR cell line authentication in 2014 and 2012, respectively. Both these cell lines passed mycoplasma test. Furthermore, the 9-STR profile of U251 from CBTC-CCAS matches the U-251 MG from the source of JCRB (see Supplementary Table 1 in Hu et al. 2013 [12])

The human glioblastoma cell lines of A172, LG11, LN229, T98G, and U251HF, and cervical cancer cell line Hela were from Departments of Neuro-Oncology and Biological Chemistry, the University of Texas M.D. Anderson Cancer Center in year 2003. Human prostate cancer cell lines of WPMY-1 and PC-3 were from the Dan Mercola Lab of UC Irvine in year 2008. U251-NS was established in year 2011 at the UC Irvine Brain Tumor Research Laboratory, which is a clonal line of U251HF, characterized as a stem-like tumor initiating cell (STIC) [12]. U251-GFP and U251NS-RFP used in this study were established by lentivival infection of pGIPZ-Empty and pTRIPZ-Empty vectors in U251HF and U251-NS, respectively. 51A and 51B were cultured in 2010 from a glioblastoma with *EGFR* amplification, at the UC Irvine Brain Tumor Research Laboratory, with phenotypes characterized as high invasion (STIC) and tumor mass-forming cell (TMC) at passages 4 and 13, respectively, during years of 2013 to 2015, with 16-STR profile to be unique and identical between 51A and 51B (for details, see Zhou et al. [20]). 51A and 51B at similar passages were used in this study.

The 9-STR profile of U251-NS, U251-GFP and U251NS-RFP are identical to that of U251HF, which has the highest identity matching score to U251 (NCI) compared to other U251 lines from JCRB, DSMZ, CLS, and ECACC (see Supplementary Table 1 in Hu et al. 2013 [12]. Other cell lines used in this study match 100% in the 9-STR profile of those reported in ATCC, except Hela with identity matching score of 78.6%. Cell line authentications were performed by IDEXX RADIL (Columbia, MO, USA).

The above described cell lines were validated to be free of mycoplasma using MycoAlert™ PLUS Mycoplasma from Lonza Walkersville, Inc. Prior to use in this study, these cell lines were passed 2 times and re-tested for mycoplasma using Venor GeM Mycoplasma Dection Kit, PCR-based, Sigma-Aldrich (Cal# MP0025).

Cells were cultured in DMEM/F12 (all glioblastoma cells, except the STIC lines), DMEM (Hela, WPMY-1), or RPMI-1640 (PC-3), supplemented with 5-10% fetal bovine serum. 51A and U251NS-RFP cells were cultured in DMEM/F12 supplemented with epidermal growth factor (EGF, 20 ng/ml), basic fibroblast growth factor (FGF, 10 ng/ml), and 0.5% B27 (Invitrogen, Carlsbad, CA, USA). In experiments to examine the ZR30 effect on *in vitro* cultures, monolayer cultures of 51A and U251NS-RFP were achieved by culture in fibronectin (1 µg/cm^2^)-coated dishes.

### Animal use for intracranial (i.c.) GBM xenograft and intra-tumoral (i.t.) injection models

The animal protocols in this study were approved by the institutional review board. Methods of i.c. cell implantation, survival data and sample collection were described previously [12]. BALB/c nude mice (SPF level, 5-6 weeks old, female) were purchased from the Shanghai Laboratory Animal Center (SLAC), housed in a SPF level vivarium facility at the Neurosurgery & Brain and Nerve Research Laboratory, The First Affiliated Hospital of Soochow University, Suzhou, China. Human GBM cells were implanted into the right frontal lobes of nude mice via two approaches, namely by free-hand injection and cannula-guided injection as described below.

Free-hand-injection was performed for U251 and a 1:1 mixture of U251-GFP and U251NS-RFP cells. Mice were anesthetized by intraperitoneal injection of 1% sodium pentobarbital. After alcohol disinfection of the skin at the top of the head, a 0.5-1.0 cm longitudinal incision was made in the frontal area. Then the skull was carefully drilled with a needle tip 3.5 mm from the cerebral midline, 2 mm frontal to the coronal suture. A 10 µL syringe containing 3 µL cells (1 x 10^5^) was vertically inserted through the skull, into the brain parenchyma 3 mm down from the cerebral seam, and the cells were slowly injected over 2 min. 1-3 weeks later, the mouse was anesthetized, and a 10-µL syringe containing 5-10 µL ZR30 (180 ng/µL) was vertically inserted through the prior injection spot 3 mm down and slowly injected into the tumor-forming site over 2-3 min.

Cannula-guided injection was performed for a 1:9 mixture of U251-GFP and U251NS-RFP cells (1 x 10^5^ / 3 µL). The anesthetized mouse was placed in a stereotactic frame and the top of the head was cleaned with iodine solution followed by alcohol. A small circular incision was made to expose enough of the skull to put in two screws and see the bregma and lambda suture points, and two screws were placed on the far left and far right after drilling just enough to put in screws. Then a hole 3 mm in depth was drilled at a spot 3 mm right of the lambda point and 2 mm anterior to the coronal suture, and a guide cannula (5 mm pedestal, 3 mm below, single, 24G) filled with a dummy (fit 3 mm) was inserted, down 0.5 mm at a time. The cement powder and liquid were mixed and added slowly to the back of the brain and allowed to flow to the front to form the head cap. Five to seven days later, without anesthetizing the mouse, 3 µL tumor cells (1 x 10^5^) were taken up with an internal cannula (fit 3 mm), inserted into the guide cannula, and injected into the brain with the speed (1.5 µl/min) controlled by a Harvard Apparatus Model 11 Plus syringe pump. One to two weeks later, without anesthetizing the mouse, an internal cannula containing 5-10 µL ZR30 was inserted into the guide cannula, after removing the infusion dummy cannula, and injected into the tumor-forming site at a speed of 1.5 µl/min.

Mice were held in sterile cages (one or five mice per cage with or without cannula implants, respectively). For mice implanted with mixture of U251-GFP and U251NS-RFP cells, water containing doxycycline (1 mg / mL) was provided throughout the experiment, to induce RFP expression by U251NS-RFP cells. Mice were observed daily after surgery and weighed every 2-3 days to determine tumor formation based on neurological deficits and weight loss. To measure survival as the end-point, mice were followed until severe neurological deficit and/or 25-30% weight-loss and the following day was recorded as the survival date. For mice found dead, the previous day was recorded as the survival date. For comparative quantitative (CQ) PCR to measure tumor cell growth and to determine the effect of the treatment, mice in both control and treatment groups were euthanized at the same time, the entire right hemispheres were removed, and their DNA was extracted.

### Gelatin zymography and antibodies for immunoblotting

Monolayer cells at 15-20% confluence were cultured in media with or without adding a few µL of ZR30 (180 ng/µL) until reaching 80-90% confluence, then cells were washed with PBS, and the culture was continued in basal medium (no supplement) with or without addition of ZR30 for 2 days. For the gelatin zymography assay, 2-day conditioned medium was collected after removing cells by a brief centrifugation, and the proteins were precipitated by acetone and used in the assay. For the immunoblotting assay, 2-day conditioned cells were treated with or without EGF (50 ng/ml) for 30 min prior to harvest, then whole cell lysates were extracted and immunoblotted for proteins of NOTCH1, EGFR, AKT, and pAKT (Ser473) with antibodies (rabbit, 1:1000 dilutions) from Cell Signaling (Danvers, MA, USA). The blots were washed and re-probed with ACTB (IgM-specific mouse, 1:1000 dilution) from Millipore (Temecula, CA, USA), to control equal protein loading. Detailed methods have been described previously [11]. Relative protein levels were determined by densitometry of each band using IMAGEJ 1.42 (NIHIMAGE), with untreated cells set in unity.

### Quantification of human gene expressions in human glioma xenografts

Total RNA samples were extracted from the entire right hemispheres of the brains, and about 1 µg RNA samples were converted to cDNA, diluted 20 times with 10 mM Tris.HCL (pH 7.5), then used (4 µL/each reaction) in real-time PCR. The primers specifically amplifying human genes, but not the homologues of mouse, and the corresponding standard were provided by Ziren Research LLC (Irvine, CA, USA).

### Quantification of human xenografts in mice by comparative quantitative (CQ) PCR mediated

Upon sacrifice of the mice, the entire right hemispheres of the brains were removed and DNA samples were extracted, diluted with 10 mM Tris-HCl (pH7.5), taken (10-20 ng) to quantify the copy numbers of human gene (*SPAG16*) and mouse gene (*Spag16*), RFP, and GFP, with primer mixtures and the CQ-PCR standards provided by Ziren Research (Irvine, CA, USA). The quantity of human and mouse single-copy-genes (e.g. SPAG16) and their ratio was used as the human/mouse cell ratio, and to compare the growth of the tumor in mice of the control and treatment groups. Ratio of RFP/GFP was used to refer ratio of two cell subpopulations; U251NS-RFP and U251-GFP.

### Real-time PCR parameters and PCR primers

We used a StepOne real-time PCR instrument (Applied Biosystems, Foster City, USA) to perform both CQ and qRT-PCR, with FastStart DNA MasterPLUS SYBR Green I (Roche, Indianapolis, USA) and the standard method with a two-step PCR program (95°C for 3 sec, 65°C for 30 sec) and Fast 96-Well Reaction Plates (0.1 mL) (Applied Biosystems, Foster City, USA). Primer sequences for CQ-PCR were 5’-GCAAGTGGCAATGGTGTTATC-3’ and 5’-GCTGGCACATTTAACCAGTTC-3’ for human *SPAG16*; 5’-AGCCATCTTCAACAGAGTCC-3’ and 5’-CTCTCTTGTGCTAATGGAGC-3’ for mouse Spag16; 5’-ATGGAGAGCGACGAGAGC-3’ and 5’-CGCCTTTGGTGCTCTTCATC-3’ for GFP in the pGIPZ-Vector, and 5’-AGGAGAACATGCACATGAAGC-3’ and 5’-GCCGTACATGAAGCTGGTAG-3’ for RFP in pTRIPZ.

Primer sequences for qRT-PCR were 5’-TGGTGACAAGTGGTGGAAC -3’ and 5’-CAGAAGAGAGGCTTTCGAC-3’ for *CDK4*, 5’-GTTAAGGTTGAGGAGCCTTC-3’ and 5’-TCCTCAATCCACGTATAGATG-3’ for *FOXM1*, 5’-CCAGGAAATGCTAGTGAGTC-3’ and 5’- ACTCGTAGTCAACTTTGTAGC-3’ for *IGFBP3*, 5’- ATGGATCCTGGCTTTCCC -3’ and 5’-GCTTCCAAACTTCACGCTC -3’ for MMP2, 5’-AACAAGTGGACGACGTTCC-3’ and 5’-CGTTCATGCTGTACACGCACA -3’ for *SEMA3B*, 5’-CCTGGTGAAAGCTGTCATCG -3’ and 5’-TGTGCCATCTCAATCTCTCG-3’ for *PTK2*, and 5’-TCCTTCCTGGGCATGGAGT -3’ and 5’-TGATCTTCATTGTGCTGGGT -3’ for *ACTB.*

### Statistical analysis

Overall survival of mice bearing intracranial GBM xenografts was estimated using Kaplan–Meier survival curves, and *P* values were determined from Wilcoxon pair-wise and Log-Rank statistics on pair-wise comparisons of the two groups using Cox Regression. The significance level was set at *P* < 0.01 to adjust for the multiple comparisons without overinflating Type II error. Welch’s t-test (2-tailed, unequal-variance) was performed for comparison of treatment effects on cell growth *in vivo* and gene expressions. SAS versions 9.2 and 9.3 (The SAS Institute, Cary, NC) were used for all analyses.
